# Evaluation of PET and laparoscopy in STagIng advanced gastric cancer: a multicenter prospective study (PLASTIC-study)

**DOI:** 10.1186/s12885-018-4367-9

**Published:** 2018-04-20

**Authors:** H. J. F. Brenkman, E. C. Gertsen, E. Vegt, R. van Hillegersberg, M. I. van Berge Henegouwen, S. S. Gisbertz, M. D. P. Luyer, G. A. P. Nieuwenhuijzen, J. J. B. van Lanschot, S. M. Lagarde, W. O. de Steur, H. H. Hartgrink, J. H. M. B. Stoot, K. W. E. Hulsewe, E. J. Spillenaar Bilgen, M. J. van Det, E. A. Kouwenhoven, D. L. van der Peet, F. Daams, J. W. van Sandick, N. C. T. van Grieken, J. Heisterkamp, B. van Etten, J. W. Haveman, J. P. Pierie, F. Jonker, A. Y. Thijssen, E. J. T. Belt, P. van Duijvendijk, E. Wassenaar, H. W. M. van Laarhoven, F. J. Wessels, N. Haj Mohammad, H. F. van Stel, G. W. J. Frederix, P. D. Siersema, J. P. Ruurda, on behalf of the PLASTIC Study Group

**Affiliations:** 1 Department of Surgery, University Medical Center Utrecht, Utrecht University, Heidelberglaan 100, 3584 CX Utrecht, The Netherlands; 2 The Netherlands Cancer Institute – Antoni van Leeuwenhoek, Amsterdam, The Netherlands; 3 Academic Medical Center, Amsterdam, The Netherlands; 4 Catharina Hospital, Eindhoven, The Netherlands; 5 Erasmus Medical Center, Rotterdam, The Netherlands; 6 Leiden University Medical Center, Leiden, The Netherlands; 7 Zuyderland Medical Center, Sittard-Geleen, The Netherlands; 8 Rijnstate Hospital, Arnhem, The Netherlands; 9 ZGT Hospitals, Almelo, The Netherlands; 10 VU University Medical Center, Amsterdam, The Netherlands; 11 Elisabeth Twee-Steden Hospital, Tilburg, The Netherlands; 12 University Medical Center Groningen, University of Groningen, Groningen, The Netherlands; 13 Medical Center Leeuwarden, Leeuwarden, The Netherlands; 14 Albert Schweitzer Hospital, Dordrecht, The Netherlands; 15 Gelre Ziekenhuis, Apeldoorn, The Netherlands; 16 Propsective Observational Cohort study of Oesophageal-gastric cancer Patients (POCOP) of the Dutch Upper GI Cancer Group (DUCG), Amsterdam, The Netherlands; 17 Radboud University Medical Center, Nijmegen, The Netherlands

**Keywords:** Gastric cancer, Gastrectomy, Laparoscopy

## Abstract

**Background:**

Initial staging of gastric cancer consists of computed tomography (CT) and gastroscopy. In locally advanced (cT3–4) gastric cancer, fluorodeoxyglucose positron emission tomography with CT (FDG-PET/CT or PET) and staging laparoscopy (SL) may have a role in staging, but evidence is scarce. The aim of this study is to evaluate the impact and cost-effectiveness of PET and SL in addition to initial staging in patients with locally advanced gastric cancer.

**Methods:**

This prospective observational cohort study will include all patients with a surgically resectable, advanced gastric adenocarcinoma (cT3–4b, N0–3, M0), that are scheduled for treatment with curative intent after initial staging with gastroscopy and CT. The modalities to be investigated in this study is the addition of PET and SL. The primary outcome of this study is the proportion of patients in whom the PET or SL lead to a change in treatment strategy. Secondary outcome parameters are: diagnostic performance, morbidity and mortality, quality of life, and cost-effectiveness of these additional diagnostic modalities. The study recently started in August 2017 with a duration of 36 months. At least 239 patients need to be included in this study to demonstrate that the diagnostic modalities are break-even. Based on the annual number of gastrectomies in the participating centers, it is estimated that approximately 543 patients are included in this study.

**Discussion:**

In this study, it is hypothesized that performing PET and SL for locally advanced gastric adenocarcinomas results in a change of treatment strategy in 27% of patients and an annual cost-reduction in the Netherlands of €916.438 in this patient group by reducing futile treatment. The results of this study may be applicable to all countries with comparable treatment algorithms and health care systems.

**Trial registration:**

NCT03208621. This trial was registered prospectively on June 30, 2017.

## Background

Gastric cancer is the fifth most common type of cancer worldwide [1]. In Western countries, curative treatment consists of gastrectomy with perioperative chemotherapy [2–4]. Unfortunately, the prognosis of patients who undergo curative treatment remains relatively poor, with a 5-year overall survival rate of 20–40%. The main cause for this poor prognosis is tumor recurrence [2, 5]*.* The poor prognosis, treatment-related morbidity and mortality, and impairments in quality of life result in a high disease burden [6].

The standard diagnostic work-up of patients with gastric cancer includes a gastroscopy to assess tumor size and location and to obtain tissue to characterize the tumor. Furthermore, computed tomography (CT) of the thorax and abdomen is performed to detect metastases and evaluate local resectability. However, the accuracy of CT for detecting metastatic disease (M1) or local irresectability (T4b) is low: the sensitivity to detect peritoneal metastases is 22%–33%, to detect distant metastases is 14%–65% and to detect T4b disease is 5%–69% [7–10]. Consequently, two undesirable situations may occur in practice:

1. Unexpected intraoperative peritoneal metastases or local tumor irresectability are found at the onset of gastrectomy.

2. Undetected distant metastases presenting shortly after treatment with curative intent (neoadjuvant chemotherapy and/or surgery).

In both situations, patients undergo a futile treatment, probably leading to a reduced quality of life and an increase in health care costs.

A recent study from the United States investigated the additional staging capacities of fluorodeoxyglucose positron emission tomography with CT (PET) and staging laparoscopy (SL) in gastric cancer. In this study, combination of PET and SL identified additional metastases in 27% of patients: distant metastases by PET in 10% of patients, and peritoneal metastases by SL in 19% of patients (with an overlap of 2%) [11]*.*To reduce the number of patients undergoing futile treatment, the new Dutch guidelines for the treatment of gastric cancer recently included PET and SL in the staging algorithm of locally advanced (cT3–4) tumors“(http://www.oncoline.nl/maagcarcinoom)”. However, this guideline concludes that the evidence for both staging modalities is weak, and additional studies are needed to further investigate the cost-effectiveness and applicability of routinely adding PET and SL to the staging of locally advanced gastric cancer.

### Aim of the study

The aim of this study is to evaluate the clinical impact and cost-effectiveness of PET and SL in addition to initial staging by CT and gastroscopy in patients with locally advanced (cT3–4) gastric cancer.

## Methods

### Objectives

The primary outcome of this study is the proportion of patients in whom PET and SL lead to a change in treatment strategy. The accuracy of each modality will be analyzed separately. Secondary outcome parameters are diagnostic performance (sensitivity, specificity, positive and negative predictive value), morbidity and mortality, quality of life, cost reduction and cost-effectiveness. The hypothesis of this study is that adding PET and SL to additional staging in these patients will lead to a change of treatment strategy in 27% of patients, leading to an annual cost reduction in the Netherlands of €916.438 by reducing futile treatment.

### Study design

The study design is a prospective observational cohort study. All patients with a locally advanced tumor who are candidates for gastrectomy with curative intent will be invited to participate in this study. A locally advanced tumor is defined as a transmural tumor invading the outer layer of the stomach (cT3–4 according to the 7th edition of the American Joint Committee on Cancer TNM staging system [12]), objectified on CT [13].

### Study population

The study population consists of patients with a surgically resectable, advanced gastric adenocarcinoma (cT3–4b, N0–3, M0), who are scheduled for treatment with curative intent after initial staging with gastroscopy and CT. Patients’ inclusion and exclusion criteria are defined as follows:

Inclusion criteria:

• Histologically proven adenocarcinoma of the stomach or esophagogastric junction (Siewert type III) as observed by gastroscopy.

• Underwent evaluation with CT of the abdomen and chest.

• Surgically resectable, advanced gastric cancer (cT3–4b, N0–3, M0), as determined by the multidisciplinary team (MDT).

• Intention to perform a potentially curative gastrectomy with or without perioperative treatment.

Exclusion criteria.

• Siewert type I-II esophagogastric junction tumor.

• Unfit or unwilling to undergo surgery.

### Study protocol

#### Initial staging

Initial staging should be performed according to national guidelines, including at least gastroscopy with tumor biopsies and a CT of the thorax and abdomen. Endoscopic Ultrasonography (EUS) may be performed optionally. In case of a cT3–4 tumor (defined as a transmural tumor invading the outer layer of the stomach) [13], patients will be invited to participate in this study and thereby give permission to collect and analyze their data. Differentiation between cT2 and cT3 tumors is not always possible with initial staging. In case of considerable doubt whether a tumor is cT2 or cT3, patients will be included if deemed appropriate by the MDT. As part of a side study, an expert panel will review all CT-scans to reach consensus on the clinical T-stage.

#### Patient inclusion

If eligible for treatment with curative intent by the MDT, patients will be invited to participate in this study. Patients will be informed and included at the outpatient department of one of the Dutch investigational centers or its associated hospitals. As this study does not allocate patients to study interventions other than usual care, as recommended by the new Dutch guidelines, this study does not fall within the Medical Research Involving Human Subjects Act (WMO). Patients will be asked to sign informed consent form to confirm that they know that their data will be anonymously used for research purposes, and approve to fill out quality of life questionnaires, making use of the previously reported infrastructure of POCOP [14].

#### Investigated modalities

**Fig. 1:**
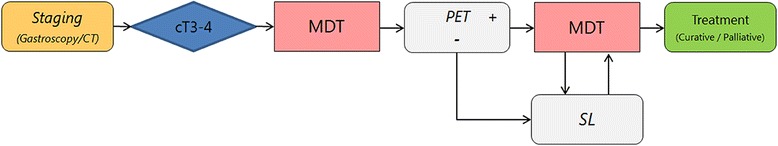
Study Flowchart. CT: computed tomography; cT3–4: advanced tumor with clinical T-stage 3 or 4.; MDT: Multidisciplinary Team; PET: fluorodeoxyglucose positron emission tomography with CT; SL: Staging Laparoscopy

The modalities to be investigated in this study are both PET and SL in addition to the initial staging with gastroscopy and CT of patients with an advanced gastric cancer (cT3–4). Patients will undergo PET and SL according to the recently revised Dutch guidelines [15]. All patients will first undergo a PET, and if the PET does not show evidence of distant metastases a SL will be performed (Fig. 1). PET or SL may be omitted if it is deemed appropriate by the MDT or if it appears that a patient is not able to undergo one of both modalities.

#### FDG-pet/CT

Preparation of patients for PET, and scanning and image reconstruction will be performed according to the institutional protocols of the participating centers, preferably incorporating guidelines of the European Association of Nuclear Medicine (EANM) / EANM Research Ltd. (EARL) and/or Netherlands Association of Nuclear Medicine (NVNG) [16]. In general, patients should refrain from strenuous exercise, and fast for at least 4 to 6 h before the injection of FDG. Patients should be pre-hydrated by drinking approximately 1 L of water in the 2 h before injection. Fasting blood glucose should preferably be below 11 mmol/L. After the injection of FDG, patients will remain seated or lying, and silent for 1 h in a warm room. A full body PET scan will be performed 60 min (range 55–75 min) after the injection of FDG, accompanied by a CT at the same scanning range. Scans are read, interpreted and reported by the nuclear medicine physicians of the respective participating centers. The report generally includes information regarding the FDG-avidity of the primary tumor and/or locoregional lymph nodes, and suspicion of distant metastases. For this study, the maximum standardized uptake values (SUVmax, corrected for body weight) of the primary tumor will also be registered. If the PET identifies new lesions that are possible metastases, a histological of cytological biopsy and/or additional imaging of a lesion is advised to confirm or exclude metastases.

#### Staging laparoscopy

SL will be performed after the PET, prior to the initiation of treatment, and should be performed by or under supervision of a gastrointestinal or oncological surgeon. During staging laparoscopy, the goals are to evaluate the resectability of the primary tumor (T-stage) and to evaluate the presence or absence of peritoneal metastases. To evaluate the resectability of the tumor, a thorough inspection of the region of the stomach and tumor along with surrounding organs will be performed. In case of a tumor localized at the posterior wall of the stomach, it is advised to open the omental bursa and inspect it accordingly. To evaluate the presence or absence of peritoneal metastases, all quadrants of the peritoneal cavity and Douglas’ pouch will be inspected. In case of suspicious macroscopic lesions, biopsies will be taken and sent for histological review. When macroscopic lesions are present, all thirteen regions of the abdomen will be evaluated and the peritoneal cancer index (PCI) will be scored [17]. Cytology of the peritoneal cavity should be performed (500cm^3^ dispersed throughout all quadrants and Douglas’ pouch) as it is a promising prognostic factor with possible implications for treatment in the future [18, 19].

#### MDT

Ideally, patients will be discussed in a first MDT after initial staging with gastroscopy and CT of the thorax and abdomen, and in a second MDT after additional staging with PET and SL (Fig. 1). In practice, in some patients the first or second MDT will be skipped and patients will proceed to additional staging or treatment without intervention of MDT’s. An included patient should be discussed in at least one MDT. Occasionally, an additional diagnostic modality will be required and the patient is discussed during a third MDT.

#### Treatment

If the tumor is deemed to be resectable, patients will be scheduled for treatment. Treatment will not be initiated before completion of staging according to the Dutch guidelines, including PET and SL [15]. There are no additional restrictions to the further treatment strategy, such as chemotherapy regimen or type/approach of resection.

### Outcome measurements

The primary outcome of this study is the proportion of patients in whom PET and/or SL leads to a change in treatment strategy. This includes the proportion of patients in whom surgery with curative intent is prevented, and the proportion of patients in whom the chemotherapy regimen is changed or omitted. Secondary outcome parameters include cost-effectiveness, modality-specific performances (diagnostic performance of both modalities, incidental findings on PET), patients’ extra burden of the diagnostic modalities (morbidity and mortality, diagnostic delay, number of extra MDT’s held), and overall quality of life of patients (EORTC Quality of Life questionnaires).

### Sample size calculation

Based on previous literature it is expected that 27% of patients will have a change in treatment strategy [11]. Taking a safety margin of 5% into account (thus at least 22% of patients will have a change in treatment strategy), an alpha of 0.05 and a power of 0.80, at least 239 patients need to be included in this study to demonstrate that performing a combination of both diagnostic modalities is cost-effective. Based on the yearly number of gastrectomies performed in the participating centers, approximately 543 patients are expected to be eligible for the study in 36 months.

### Statistical analysis

**Fig. 2:**
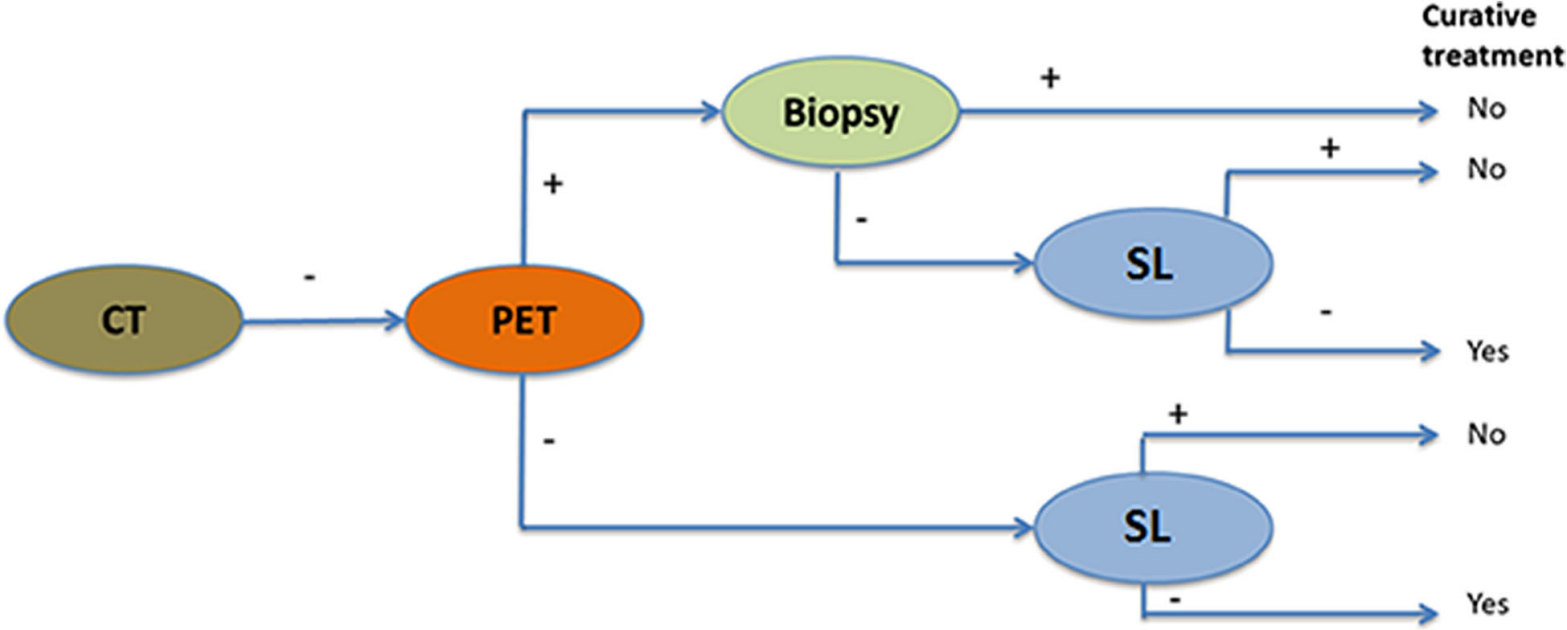
Decision tree for PET and SL. CT: computed tomography; PET: fluorodeoxyglucose positron emission tomography with CT; SL: Staging Laparoscopy

The primary outcome measures, change in treatment strategy of PET and SL, will be presented as a percentage. To evaluate the performance of PET and SL, sensitivity and specificity will be calculated. A separate analysis will be performed to assess whether both diagnostic modalities are accurate for various subgroups, for instance tumor types (Lauren classification: diffuse, intestinal and mixed). The quality of life of patients in this study will be compared to previous data from literature and to a retrospective cohort of patients who did not undergo PET and SL. Differences are tested using linear mixed-effects modeling, taking relevant patient characteristics into account. Missing values will be imputed using multiple imputation techniques. Statistical significance is defined as *p* < 0.05. Cost-effectiveness will be calculated, taking all relevant health-related costs into account, including costs arising from complications of medical treatment, and additional diagnostics arising from false-positive findings on PET. A model (Fig. 2) will be developed to compare health care costs with only CT. A budget impact analysis (BIA) will be performed, adhering to the newest guidelines and applying the societal, health insurance/third party payer and health care perspectives. Analyses will be performed for the combination of PET and SL and for both treatment modalities separately.

### Time schedule

The study recently started on 01–08-2017, and will last for 36 months. After the start of the study, the first 30 months will consist of inclusion and follow-up of the patients. The last 6 months will consist of follow-up and analysis of results. The study will end at 01–08-2020.

## Discussion

The PLASTIC study is a prospective observational cohort study evaluating the impact and cost-effectiveness of PET and SL in addition to initial staging by CT and gastroscopy in patients with locally advanced gastric cancer. The new Dutch guidelines recently included these staging modalities for staging locally advanced gastric cancer but recommended additional studies to be performed.

Until recently, the role of PET in the staging of gastric cancer has been limited. Indeed, during initial staging of gastric cancer, the sensitivity and specificity of PET are not better than that of CT for lymph node metastases, liver metastases and peritoneal metastases [7–9, 15]. However, for patient with locally advanced tumors, PET may be able to find additional distant metastases which were not detected during initial staging with CT. This was recently reported in a single study from the United States by Smyth et al., who prospectively evaluated the utility of PET and SL in patients with locally advanced gastric cancer [11]. They found that PET is able to detect additional distant metastases in 10% of patients, resulting in an estimated cost reduction of $13.000 per patient. Moreover, they concluded that PET is most cost-effective if performed prior to SL [11]. However, the cost-effectiveness analysis was performed retrospectively and might not be applicable to health care systems outside the US.

SL prior to gastrectomy has been broadly applied to diagnose peritoneal metastases. Studies evaluating the percentage of patients who benefit from SL are abundant, with percentages varying between 16 and 38% [10, 20–27]. Unfortunately, high quality evidence supporting cost-effectiveness is not available. The PLASTIC study will be the first study to prospectively investigate the cost-effectiveness of SL in combination with PET.

In this study, patients will first undergo a PET, followed by a SL if PET does not show distant metastases. If distant metastases found on PET are confirmed by biopsy or additional imaging, SL will be omitted. This order was chosen as it is more applicable in clinical practice for the following reasons:

- PET is non-invasive, whereas a SL is invasive and is accompanied by a higher risk of adverse events for the patients.
- Assessing the PET after SL will be less reliable, due to uptake of FDG in postoperative inflammation.
- PET scans can be scheduled more easily and probably results in less diagnostic delay.
- In a theoretical model from a previous study, first performing PET resulted in more cost savings compared to a SL-first approach (difference $2168 per patient) [11].

This study aims to include patients with locally advanced (cT3–4) gastric cancer, but the accuracy of CT for determining T-stage is low [8]. This could result in the unintended inclusion of early stage (T2) tumors or failure to include truly advanced tumors. Nevertheless, these flaws reflect current practice. To reduce the impact of these current limitations, an expert panel will review all CT-scans to reach consensus on the clinical T-stage as a side study.

The current study is relevant, as the addition of PET and SL may prevent futile gastrectomies, which are associated with considerable morbidity, mortality, reduction in quality of life and costs [28–30]. On the other hand, the number of prevented gastrectomies should exceed a certain level, as PET and SL are accompanied by costs and possible risks for the patient (ionizing radiation, surgical complications) as well. The results of this study will be implemented in an updated version of the Dutch guidelines, but may also be applicable to all other Western countries with comparable treatment algorithms and health care systems.

By performing staging laparoscopy before starting treatment, a side effect of this study might be that more patients with peritoneal carcinomatosis will be detected. Recent studies have shown that there may be a role for hyperthermic intraperitoneal chemotherapy (HIPEC) and cytoreductive surgery in these patients [31, 32]. Patients with peritoneal carcinomatosis as detected in this study may therefore be included in HIPEC trials [33].

This prospective observational cohort study will evaluate the impact and cost-effectiveness of FDG-PET/CT and staging laparoscopy in addition to initial staging by CT and gastroscopy in patients with locally advanced gastric cancer. It is hypothesized that in 27% of patients a change in treatment strategy will occur, and that the annual cost reduction in the Netherlands will be approximately €916.438.

### Trial status

As this study does not fall under the Medical Research Involving Human Subjects Act (WMO), a waiver for ethical approval (16–633/C) was obtained from the ethical review board of the UMC Utrecht. Recruitment of patients started in August 2017.

## Abbreviations

BIA: Budget impact analysis
CT: Competed tomography
EANM: European association of nuclear medicine
EARL: EANM Research Ltd.
EUS: Endoscopic ultrasonography
FDG: Fluorodeoxyglucose
FDG-PET/CT: Fluorodeoxyglucose positron emission tomography
HIPEC: Hyperthermic intraperitoneal chemotherapy
MDT: Multidisciplinary team
NVNG: Netherlands association of nuclear medicine
PCI: Peritoneal cancer index
PET: Positron emission tomography
PLASTIC: Evaluation of PET and laparoscopy in STagIng advanced gastric cancer: a multicenter prospective study
POCOP: Prospective observational cohort study of oesophageal-gastric cancer patients
SL: Staging laparoscopy
SUVmax: Maximum standardized uptake values
WMO: Medical research involving human subjects act
ZonMW: Netherlands organization for health research and development

## References

[1] Ferlay J, Soerjomataram I, Dikshit R, Eser S, Mathers C, Rebelo M, Parkin DM, Forman D, Bray F (2015). Cancer incidence and mortality worldwide: sources, methods and major patterns in GLOBOCAN 2012. Int J Cancer.

[2] Cunningham D, Allum WH, Stenning SP, Thompson JN, Van de Velde CJ, Nicolson M, Scarffe JH, Lofts FJ, Falk SJ, Iveson TJ, Smith DB, Langley RE, Verma M, Weeden S, Chua YJ, MAGIC Trial P (2006). Perioperative chemotherapy versus surgery alone for resectable gastroesophageal cancer. N Engl J Med.

[3] Brenkman HJ, Haverkamp L, Ruurda JP, van Hillegersberg R (2016). Worldwide practice in gastric cancer surgery. World J Gastroenterol.

[4] Al-Batran S, Homann N, Schmalenberg H, Kopp H, Haag GM, Luley KB, Schmiegel WH, Folprecht G, Probst S, Prasnikar N, Thuss-Patience P, Fischbach W, Trojan J, Koenigsmann M, Pauligk C, Goetze TO, Jaeger E, Meiler J, Schuler MH, Hofheinz R (2017). Perioperative chemotherapy with docetaxel, oxaliplatin, and fluorouracil/leucovorin (FLOT) versus epirubicin, cisplatin, and fluorouracil or capecitabine (ECF/ECX) for resectable gastric or gastroesophageal junction (GEJ) adenocarcinoma (FLOT4-AIO): a multicenter, randomized phase 3 trial. JCO.

[5] Dassen AE, Dikken JL, Bosscha K, Wouters MW, Cats A, van de Velde CJ, Coebergh JW, Lemmens VE (2014). Gastric cancer: decreasing incidence but stable survival in the Netherlands. Acta Oncol.

[6] Hoeymans N, Melse JM, Schoemaker CG: Gezondheid en determinanten. Deelrapport van de VTV 210 Van gezond naar beter. 2010, RIVM rapport 270061006: http://www.rivm.nl/bibliotheek/rapporten/270061006.pdf.

[7] Choi JY, Shim KN, Kim SE, Jung HK, Jung SA, Yoo K (2014). The clinical value of 18F-fluorodeoxyglucose uptake on positron emission tomography/computed tomography for predicting regional lymph node metastasis and non-curative surgery in primary gastric carcinoma. Korean J Gastroenterol.

[8] Seevaratnam R, Cardoso R, McGregor C, Lourenco L, Mahar A, Sutradhar R, Law C, Paszat L, Coburn N (2012). How useful is preoperative imaging for tumor, node, metastasis (TNM) staging of gastric cancer? A meta-analysis. Gastric Cancer.

[9] Wang Z, Chen JQ (2011). Imaging in assessing hepatic and peritoneal metastases of gastric cancer: a systematic review. BMC Gastroenterol.

[10] Hu YF, Deng ZW, Liu H, Mou TY, Chen T, Lu X, Wang D, Yu J, Li GX (2016). Staging laparoscopy improves treatment decision-making for advanced gastric cancer. World J Gastroenterol.

[11] Smyth E, Schoder H, Strong VE, Capanu M, Kelsen DP, Coit DG, Shah MA (2012). A prospective evaluation of the utility of 2-deoxy-2-[(18) F]fluoro-D-glucose positron emission tomography and computed tomography in staging locally advanced gastric cancer. Cancer.

[12] Edge S, Byrd D, Compton C, Fritz A, Greene F, Trotti A (2010). AJCC cancer staging manual.

[13] Kim JW, Shin SS, Heo SH, Choi YD, Lim HS, Park YK, Park CH, Jeong YY, Kang HK (2012). Diagnostic performance of 64-section CT using CT gastrography in preoperative T staging of gastric cancer according to 7th edition of AJCC cancer staging manual. Eur Radiol.

[14] Coebergh van den Braak RRJ, van Rijssen LB, van Kleef JJ, Vink GR, Berbee M, van Berge Henegouwen MI, Bloemendal HJ, Bruno MJ, Burgmans MC, Busch ORC, Coene PPLO, Coupe VMH, Dekker JWT, van Eijck CHJ, Elferink MAG, Erdkamp FLG, van Grevenstein WMU, De Groot JWB, van Grieken NCT, de Hingh IHJT, Hulshof MCCM, Ijzermans JNM, Kwakkenbos L, Lemmens VEPP, Los M, Meijer GA, Molenaar IQ, Nieuwenhuijzen GAP, de Noo ME, van de Poll-Franse LV, Punt CJA, Rietbroek RC, Roeloffzen WWH, Rozema T, Ruurda JP, van Sandick JW, Schiphorst AHW, Schipper H, Siersema PD, Slingerland M, Sommeijer DW, Spaander MCW, Sprangers MAG, Stockmann HBAC, Strijker M, van Tienhoven G, Timmermans LM, Tjin-A-Ton MLR, van der Velden AMT, Verhaar MJ, Verkooijen HM, Vles WJ, de Vos-Geelen JMPGM, Wilmink JW, Zimmerman DDE, van Oijen MGH, Koopman M, Besselink MGH, van Laarhoven HWM, Dutch Pancreatic Cancer Group, Dutch Upper GI Cancer Group and PLCRC working group.: Nationwide comprehensive gastro-intestinal cancer cohorts: the 3P initiative. Acta Oncol. 2018;57(2):195–202. 10.1080/0284186X.2017.1346381 28723307

[15] IKNL (2015). Richtlijn Maagcarcinoom conceptversie.

[16] Boellaard R, Delgado-Bolton R, Oyen WJ, Giammarile F, Tatsch K, Eschner W, Verzijlbergen FJ, Barrington SF, Pike LC, Weber WA, Stroobants S, Delbeke D, Donohoe KJ, Holbrook S, Graham MM, Testanera G, Hoekstra OS, Zijlstra J, Visser E, Hoekstra CJ, Pruim J, Willemsen A, Arends B, Kotzerke J, Bockisch A, Beyer T, Chiti A, Krause BJ (2015). European Association of Nuclear Medicine (EANM): FDG PET/CT: EANM procedure guidelines for tumour imaging: version 2.0. Eur J Nucl Med Mol Imaging.

[17] Sugarbaker PH (1998). Management of peritoneal surface malignancy using intraperitoneal chemotherapy and cytoreductive surgery: manual for physicians and nurses.

[18] Japanese Gastric Cancer Association (2011). Japanese classification of gastric carcinoma: 3rd English edition. Gastric Cancer.

[19] Jamel S, Markar SR, Malietzis G, Acharya A, Athanasiou T, Hanna GB (2017). Prognostic significance of peritoneal lavage cytology in staging gastric cancer: systematic review and meta-analysis. Gastric Cancer.

[20] Tourani SS, Cabalag C, Link E, Chan ST, Duong CP (2015). Laparoscopy and peritoneal cytology: important prognostic tools to guide treatment selection in gastric adenocarcinoma. ANZ J Surg.

[21] Santa-Maria AF, Valadao M, Iglesias AC (2014). The role of staging laparoscopy in treatment of locally advanced gastric cancer. Surg Laparosc Endosc Percutan Tech.

[22] Huang H, Jin JJ, Long ZW, Wang W, Cai H, Liu XW, Yu HM, Zhang LW, Wang YN (2014). Three-port laparoscopic exploration is not sufficient for patients with T4 gastric cancer. Asian Pac J Cancer Prev.

[23] Kapiev A, Rabin I, Lavy R, Chikman B, Shapira Z, Kais H, Poluksht N, Amsalam Y, Halpern Z, Markon I, Wassermann I, Halevy A (2010). The role of diagnostic laparoscopy in the management of patients with gastric cancer. Isr Med Assoc J.

[24] Mahadevan D, Sudirman A, Kandasami P, Ramesh G (2010). Laparoscopic staging in gastric cancer: an essential step in its management. J Minim Access Surg.

[25] Muntean V, Mihailov A, Iancu C, Toganel R, Fabian O, Domsa I, Muntean MV (2009). Staging laparoscopy in gastric cancer. Accuracy and impact on therapy. J Gastrointestin Liver Dis.

[26] Shelat VG, Thong JF, Seah M, Lim KH (2012). Role of staging laparoscopy in gastric malignancies - our institutional experience. World J Gastrointest Surg.

[27] Ikoma N, Blum M, Chiang YJ, Estrella JS, Roy-Chowdhuri S, Fournier K, Mansfield P, Ajani JA, Badgwell BD (2016). Yield of staging laparoscopy and lavage cytology for radiologically occult peritoneal Carcinomatosis of gastric Cancer. Ann Surg Oncol.

[28] Brenkman HJ, Ruurda JP, Verhoeven RH, van Hillegersberg R (2017). Safety and feasibility of minimally invasive gastrectomy during the early introduction in the Netherlands: short-term oncological outcomes comparable to open gastrectomy. Gastric Cancer.

[29] Busweiler LA, Wijnhoven BP, van Berge Henegouwen MI, Henneman D, van Grieken NC, Wouters MW, van Hillegersberg R, van Sandick JW (2016). Dutch upper gastrointestinal Cancer audit (DUCA) group: early outcomes from the Dutch upper gastrointestinal Cancer audit. Br J Surg.

[30] Avery K, Hughes R, McNair A, Alderson D, Barham P, Blazeby J (2010). Health-related quality of life and survival in the 2 years after surgery for gastric cancer. Eur J Surg Oncol.

[31] Glehen O, Gilly FN, Arvieux C, Cotte E, Boutitie F, Mansvelt B, Bereder JM, Lorimier G, Quenet F, Elias D (2010). Association Francaise de Chirurgie: peritoneal carcinomatosis from gastric cancer: a multi-institutional study of 159 patients treated by cytoreductive surgery combined with perioperative intraperitoneal chemotherapy. Ann Surg Oncol.

[32] Desiderio J, Chao J, Melstrom L, Warner S, Tozzi F, Fong Y, Parisi A, Woo Y (2017). The 30-year experience-a meta-analysis of randomised and high-quality non-randomised studies of hyperthermic intraperitoneal chemotherapy in the treatment of gastric cancer. Eur J Cancer.

[33] van der Kaaij RT, Braam HJ, Boot H, Los M, Cats A, Grootscholten C, Schellens JH, Aalbers AG, Huitema AD, Knibbe CA, Boerma D, Wiezer MJ, van Ramshorst B, van Sandick JW (2017). Treatment of peritoneal dissemination in stomach Cancer patients with Cytoreductive surgery and Hyperthermic intraperitoneal chemotherapy (HIPEC): rationale and design of the PERISCOPE study. JMIR Res Protoc.

